# Image-Guided Proton Therapy: A Comprehensive Review

**DOI:** 10.3390/cancers15092555

**Published:** 2023-04-29

**Authors:** Shelby A. Lane, Jason M. Slater, Gary Y. Yang

**Affiliations:** James M. Slater, MD Proton Treatment and Research Center, Loma Linda University, Loma Linda, CA 92354, USAgyang@llu.edu (G.Y.Y.)

**Keywords:** image guidance, proton therapy, dose guided RT, MRI guided PT, surface guided PT, FLASH, upright proton treatment

## Abstract

**Simple Summary:**

In proton therapy, there is a sharp peak in the delivered dose followed by a rapid falloff, known as the Bragg peak, which is not present in photons. This allows for treatment plans that deliver lower doses to normal tissue than can be performed with photons. This requires a high degree of accuracy and precision of delivery due to the short distance between an area of high and low doses. Image guidance allows for better visualization of the target and more accurate delivery of proton (and photon) radiation. The equipment used to deliver proton therapy differs in several ways from that of photon radiation, which impacts the methods used for image guidance in proton therapy. This paper aims to summarize the various methods of image guidance in current proton therapy and their relative advantages and disadvantages, as well as areas for future improvements.

**Abstract:**

Image guidance for radiation therapy can improve the accuracy of the delivery of radiation, leading to an improved therapeutic ratio. Proton radiation is able to deliver a highly conformal dose to a target due to its advantageous dosimetric properties, including the Bragg peak. Proton therapy established the standard for daily image guidance as a means of minimizing uncertainties associated with proton treatment. With the increasing adoption of the use of proton therapy over time, image guidance systems for this modality have been changing. The unique properties of proton radiation present a number of differences in image guidance from photon therapy. This paper describes CT and MRI-based simulation and methods of daily image guidance. Developments in dose-guided radiation, upright treatment, and FLASH RT are discussed as well.

## 1. Introduction

Image guidance for external beam radiation therapy has revolutionized the field of radiation oncology. Image guidance is the use of imaging at the pretreatment or treatment stage that leads to an action that improves or verifies the accuracy of radiotherapy. Due to the Bragg peak, proton therapy (PT) is able to deliver a highly conformal dose to a target; however, the Bragg peak must be correctly placed in the target in order to utilize the potential of proton therapy. Compared to photons, protons have additional uncertainties in the range, or penetration, of the beam in tissue; these uncertainties are predominately influenced by the densities of tissue through which the beam passes. Image guidance (along with proper immobilization) minimizes these uncertainties and illustrates the greater importance it has on proton therapy compared to photon therapy. Further illustrating this point is that when the first hospital-based proton therapy center began operation in 1990, image guidance was used for every fraction of treatment that was delivered [1].

The patient must be positioned properly prior to treatment. This can be achieved by planar X-rays, CT scanners, and visual-based methods. There is also increased interest in using MRIs for positioning, although this has some challenges. The target may also be monitored during the treatment for motion. There are differences in the ways that image guidance is used for protons vs. photons due to the intrinsic properties of protons as well as the differences in hardware between linear accelerators and proton gantries. Dose-guided radiation therapy seeks to spatially measure doses from the byproducts of proton radiation. Finally, FLASH radiotherapy, an ultrahigh dose rate method of external beam radiation, which will involve predominantly protons, has its own set of unique issues for image guidance that will be mentioned.

## 2. Imaging for Simulation

Different methods of imaging have differing benefits and drawbacks when used for simulation (See Table 1).

### 2.1. CT-Based Simulation

One key difference between protons and photons when using CT scans for planning is the additional step of deriving proton-stopping power from Hounsfield Units (HU). This process of deriving the actions of the charged proton from the actions of the chargeless, massless X-ray photon leads to greater range uncertainty, roughly 3.5% of the absolute range [2,3]. Additional margins have to be added to target volumes to compensate, decreasing the therapeutic benefit of protons.

Dual-energy CT can be used to better differentiate the composition of materials [4] and further reduce the range uncertainty [5,6]. Another solution is the use of proton CT, which allows for direct reconstruction of proton stopping power, although these are currently not in widespread use due to issues with spatial resolution [7]. One group has compared images from a clinically realistic proton CT scanner to those from photon CT scanners and found proton-stopping power measurement discrepancies of up to 40% in regions with mixed content of air, soft tissue, and bone, such as sinuses [8]. A comparison between a dual-energy CT scanner and a proton CT prototype in calculating relative stopping power (RSP) of phantoms of a known RSP showed that both scanners were accurate to within 1%, that the proton CT was slightly more accurate than the dual-energy CT scanner, despite characteristic artifacts reducing the accuracy of the proton CT [9].

### 2.2. MRI-Based Simulation

MRI-only simulation for treatment planning has also been explored. One benefit of this approach is the superior contrast with MRI when compared to CT. This also may eliminate the need for registration of an MRI to a CT simulation, which introduces additional uncertainty, especially when an MRI is obtained at a different timepoint or in a different position from the simulation CT. Possible drawbacks include an initial unfamiliarity with performing MRI simulation as well as possible incompatibility and artifacts with metallic hardware such as older pacemakers [10].

Another drawback of MRI-only simulation relevant for both proton-based (as well as photon-based) treatment is a possible geometric distortion of images due to changes in the magnetic field uniformity from both the MR system as well as the patient’s body itself. An excellent review by Schmidt and Payne goes into further detail [11].

The main challenge for implementing MRI-only simulation is that the MRI data needs to be converted into a pseudo-CT dataset for patient setup and dose calculation. One strategy includes assigning electron density values to predefined tissue levels [12,13]. Another involves the creation of an MR-to-CT image atlas, with the registration of the patient’s MRI to the atlas and conversion of the MRI intensity to HU values [14]. Each comes with drawbacks, which makes them not ideal for MRI-only simulation use [15].

More recently, there has been interest in developing methods of automated planning in order to improve the workflow and reliability of MR-based planning [13]. One group has used a machine learning-based method to generate pseudo-CT images from pretreatment MRIs and retrospectively analyzed the dose–volume histogram metrics from these pseudo-CTs compared to their actual plans, which were made for the CT’s acquired pretreatment. They found that this model created accurate pseudo-CT images with good agreement from their simulation CT [16]. The value of pseudo-CT in MRI-PT has yet to be validated prospectively to the authors’ knowledge.

**Table 1:** Advantages and Disadvantages of Photon CT, MRI, and Proton CT for Simulation.

Method	Advantages	Disadvantages
Photon CT	Widespread availability and familiarity of CT simulation for therapists and physicians Dual-energy CT provides additional clarification of soft tissue density	Risk of Contrast-induced Nephropathy Uncertainty with the conversion from HU to proton-stopping power
MRI	Superior soft tissue contrast to Photon CT Possibility of acquiring sequences that provide additional information about tumor biology Registration of diagnostic MRI to simulation CT is not needed	Initial unfamiliarity with performing MRI simulation Older pacemakers may not be MRI-compatible [10] Requires generation of pseudo-CT for patient setup and dose calculation
Proton CT	Direct visualization of proton-stopping power	Current lack of proton CT scanners ready for clinical use Lower-quality anatomic visualization

## 3. Imaging for Pretreatment Positioning

Various imaging modalities are used for pretreatment positioning (See Table 2), often in complement with one another.

### 3.1. 2D Kilovoltage Imaging

#### 3.1.1. Anatomical Structure

Due to the increased sensitivity of patient positioning to dosimetry, image guidance was implemented early in the history of proton therapy [5]. Proton treatment systems have kV X-ray tubes mounted in the nozzle for a beams-eye-view setup as well as orthogonal imagers, which can be used prior to volumetric imaging. These can be moved into and out of the beam path prior to treatment. Daily images can be taken during setup compared to the digitally reconstructed radiographs (DRRs) from the time of the simulation, while the corrective moves can be performed based on the bony anatomy [17], often on a robotic 6-degree-of-freedom couch [18,19,20]. One caveat is that these can lengthen the path and increase the spot size for pencil beam scanning (PBS) gantries if downstream of the beamline vacuum window. This has been avoided in some systems by placing the X-ray beams on the robotic C-arms, behind the last gantry bending magnet, or attaching them to rooms instead of gantries [21]. One of the reasons for the early adoption of orthogonal kV radiography was the poor quality of proton radiography at the time [22]. One downside of orthogonal X-ray is that there is not as much information about possible tissue changes along the beam path as volumetric imaging, which could lead to overshoot or undershooting [22]. Moreover, since the virtual proton and XR sources are not perfectly coincident with one another, the XR tube only approximates the beam’s eye view of the proton beam [23].

#### 3.1.2. Fiducial Markers

In addition to using bony anatomy for the daily setup, fiducial markers may also be used for an additional setup. The advantage of using fiducial markers is that they may be placed in soft tissue, in close proximity to the target, and may provide a better surrogate for the location of the target than bony anatomy. Intraprostatic fiducial markers are placed, which allows for daily orthogonal kV imaging for IGRT of the prostate with both passively scattered protons [24] and PBS proton therapy [25]. This allows for a two-step setup, where the couch adjustments are performed based on the bony anatomy first. Then, another adjustment is performed based on the fiducial markers, or if the displacement of the markers is unacceptably high, the patient is not treated but instead undergoes preparation for treatment (bladder filling and rectal emptying), before repeating the setup.

For uveal melanoma treatments, tantalum clips are placed around the tumor behind the globe in the operating room prior to simulation. In the simulation, there was immobilization with a rigid mask and bite block, and the eye is fixed using stimulus-directed light. Pretreatment, orthogonal kV X-rays are taken and aligned with those taken at the time of the simulation [26]. At Loma Linda University, patients are treated with partial breast irradiation with protons, which can present a challenge due to the inter- and intrafraction motion of the target. Patients lie prone supported by vacuum bags with a custom low-density foam around the breast and plastic breast cup, which provides reproducible placement of the breast and limits respiratory motion. The kV X-rays are taken pretreatment and the radio-opaque surgical clips are aligned with the DRR to determine any setup corrections [27].

However, the fiducial markers may not move exactly as the target tissue. Thus, fiducial markers have a significant effect on dosimetry in proton therapy compared to photon therapy. They may cause HU artifacts on the planning CTs, which could lead to an under or overshoot from the proton beams. Moreover, the markers placed near the end of the proton path may strike them and cause an underdosing of the target region. This effect may be greater with gold markers, when markers are placed closer to the end of the beam or when there are fewer treatment beams used [21]. A hydrogel fiducial marker has been developed for use with proton radiation and has shown negligible depth-dose perturbation in a phantom [28].

#### 3.1.3. Fluoroscopy

Fluoroscopy has been used in both pre- and intrafraction to tailor the respiratory gating parameters. Intrafraction fluoroscopy can be used to gate respiratory motion [29]. One downside to fluoroscopy is the significant dose associated with this procedure [21].

### 3.2. 3D CT Imaging

The next step in image-guided proton therapy arrived with the use of volumetric imaging. CT can provide a 3D definition of the anatomy and greater information about the location of soft tissues than 2D kV radiography. It also allows for the possibility of adaptive planning [30]. This may be especially useful in disease sites that are prone to target shifts due to tumor volume changes and weight loss, such as head and neck tumors [31].

#### 3.2.1. CT on Rails

Patients can be moved on the treatment couch on rails from the gantry to CT scanners in the treatment room. Having the CT scanner several meters away from the proton gantry at an angle reduces the interference with the treatment workflow and neutron exposure; however, the CT and couch are at risk of colliding with one another in this setup. Furthermore, a CT on rails avoids the image quality issues of the cone-beam CT (CBCT) mentioned below. The major drawback to this approach is the lack of imaging capability at this isocenter, with no repeat verification being performed after the setup correction. Further, the movement of the couch and CT will lead to additional time for image guidance [22]. At the Paul Scherrer Institute, patients are positioned and imaged outside the treatment room using the same CT scanner that is used in the simulation. Then, they are moved into the treatment room where verification X-rays are taken prior to treatment. This frees up time in the treatment room to allow for more time on the beam [32].

#### 3.2.2. Cone-Beam CT

In addition to patients or CT scanners being moved for pretreatment imaging, CBCT usage is increasing for proton therapy. There are several options for the placement of the CBCT, which have been used by different facilities. It is typically placed on the gantry, and images are acquired as the gantry rotates, similar to linear accelerators [23]. For some facilities with partial gantries and limited space in the treatment room, the CBCT hardware may be installed onto the nozzle instead of the gantry, which must be retracted while not in use. Some facilities use a CBCT mounted on a robotic C-arm as well, which allows for imaging at the treatment isocenter or off of it. Due to the design, the source-to-imager distance is greater in CBCT for proton gantries vs. photon gantries, which reduces the number of scattered photons reaching the detector [22,23].

Daily pretreatment CT imaging does increase the imaging dose received by patients. Though relatively small in magnitude compared to the treatment dose [33], there is an increasing awareness that the cumulative dose may be significant, particularly in pediatric patients due to the concern for late effects and the distribution of the radiation doses [34]. This has led some to recommend the use of fiducial markers and kV X-rays instead of CBCT for daily soft tissue visualizations in pediatric patients [35]. However, one study found that the CBCT dose could be reduced by 81–98% and remains accurate when used in the setup, based on bony anatomy [36].

There is decreased accuracy with CBCT compared to diagnostic CT due to increased scatter, field-of-view (FOV) limitations, ring artifacts, and patient size. This can lead to dose errors when used for adaptive planning [21,22]; however, with correction methods, these errors can be reduced to around 1% [37]. Synthetic CTs can be created by using a neural network to correct daily CBCT imaging errors. Then, these synthetic CTs can be used for accurate dose calculations in adaptive proton therapy [38].

### 3.3. Visual Image Guidance

#### 3.3.1. Marker-Based

Visual image guidance can be used in addition to planar or volumetric X-ray-based imaging or can be used alone. External markers can be placed at the relevant locations on the patient’s skin. One advantage of this approach is the high frequency of measurement, which is appealing for tracking mobile targets and gating delivery [21]. As above, the location of external markers does not always reflect the location of an internal target. Moreover, there needs to be consistency in the daily placement of the markers. This approach has already been used in patients for photon therapy, and reflective spheres have been placed on a breathing phantom for a scanning ion beam [39].

#### 3.3.2. Surface-Based

Surface imaging consists of 3D mapping of the patient’s surface generated by a computer. This may be especially useful for targets that have a variable shape and location with respect to bony anatomy. Another advantage to this approach is that there is no need for external marker placement. At Massachusetts General Hospital, patients underwent postmastectomy radiation with spot scanning proton therapy after localization with a surface imaging system [40]. Surface imagers can also validate the operation of couch moves and the position of the nozzle [21,41].

### 3.4. MRI Guidance

MRI-guided photon therapy has become more popular in recent years. It has superior soft tissue contrast and no moving parts for 3D imaging, which allows for real-time motion monitoring [23]. This raises the possibility of reducing the normal tissue dose with MRI guidance. PTV margin reduction studies have been performed with MRI-guided photon SBRT and have shown a decrease in acute toxicity compared to CT-guided photon SBRT [42]. Due to these advances in photon therapy, there is an interest in the possibility of using MRI guidance for proton therapy.

MRI guidance eliminates the problem of additional ionizing radiation in contrast to CT guidance [43]. The superior soft tissue resolution of MRI over CT and the possibility of real-time imaging make it an attractive option for image guidance.

Pretreatment daily MRI guidance would allow for direct visualization of tumors not easily visualized by a CT or planar X-ray.

One use of MRI for image guidance that comes to mind, given its high resolution and that there is no additional dose for daily imaging, would be for online adaptive planning. A major challenge in the use of MRI guidance for proton therapy, which does not relate to photons, is the interaction between the magnetic fields within the MRI scanner and the charged protons, which causes deflections in the beam path. Due to the number of corrections that need to be made to place the Bragg peak at the same location for adaptive planning based on real-time MRI (changes in scan setting due to the magnetic field, changes in energy due to change in path length), pencil-beam scanning would be the only choice [44].

MRIs do not provide direct information on the stopping power, which can limit the feasibility of the online adaptive MRI-PT. Weekly offline on-treatment MRIs have been shown to improve plan quality for pediatric patients undergoing intensity-modulated proton therapy (IMPT) for CNS malignancies [45]. It has been shown that dose calculations based only on daily MRIs converted to synthetic CTs are feasible [15].

There have been several recent advances in making MRI-guided radiation therapy ready for clinical use. Proton gantries with MRI capabilities are currently under construction. Studies have been conducted that investigate the feasibility of MRI-only workflows [44]. There have been investigations into the hardware required to deliver MRI-guided PT, including magnet design, radiofrequency shielding requirements, rotation of the couch and gantry, measurement of the beam in the setting of strong magnetic fields, and correction of the beam [44]. One group has performed work to account for beam deflections from the fringe and imaging magnetic fields by using gantry angle offset and PBS nozzle skew along with patient-specific optimization within a PBS system [46].

## 4. Real-Time Imaging and Dose-Guided RT

### 4.1. PET

One of the limitations in PET-based range verification in protons is the difference between the Bragg peak and the PET signal.

In contrast to photon-based external beam radiation therapy, which can be measured with electronic portal imaging devices (EPIDs) after the beam has passed through the patient, the protons stop in the patient, making direct detection of the dose impossible. However, proton radiation produces byproducts such as positron and gamma radiation as it interacts with matter in a patient. Measurement of these byproducts in space and time allows for the location of the dose and is an area of ongoing research. Online monitoring of the dose may reduce range uncertainty and allow for further reduced margins, decreasing the dose to normal tissues even more. This may also allow for a better ability to determine when a re-simulation scan needs to be performed [47].

The challenges, which include the registration issues with the positioning errors in offline imaging and the cost and time demands for the development of the positron emission tomography (PET) systems, designed specifically for on-beam measurement of dose in vivo, have led to a limited amount of clinical use at this time [48]. A recent study [49] conducted at the National Center of Oncological Hadrontherapy (CNAO, Pavia, Italy) analyzed the in-beam PET data generated from eight patients treated with proton therapy at their center. They found that the standard deviation of interfraction PET activity profiles was 2.3–2.5 mm for patients without anatomical changes during treatment. Furthermore, large variations in PET range data correlated well with areas of anatomical change found on CT scans taken during the treatment course, suggesting that interfraction PET data can be used to detect morphological changes that may have a significant impact on dosimetry.

Positronium atoms consist of a positron bound to an electron. Roughly 40% of the positron annihilations in vivo occur via the formation of positronium atoms [50]. Para-positronium decays into two photons, while ortho-positronium decays into three photons. The rate at which these three-photon decays happen is proportional to the level of oxygen concentration. Therefore, positronium imaging may allow for the identification of hypoxic tissues [48]. A new PET detection system may allow for both range monitoring [51] as well as positronium imaging [52].

### 4.2. Prompt Gamma

Prompt gamma waves are produced instantaneously and have almost no interaction with the patient as they exit [21]. Detecting these gamma waves may allow for the precise location of the Bragg peak in three dimensions and the determination of the dose online in real time. Several groups are developing systems for prompt gamma detectors but there has not currently been any widespread adoption of prompt gamma detectors in patients [53]. One system is the use of a slit camera detector system, whereby prompt gamma radiation is emitted along the proton radiation path and passes through a tungsten slit collimator below the patient onto a floor-based segmented detector, which allows for spatial resolution of the prompt gamma distribution. The absolute range of protons can be determined from this distribution. This is compared to a reference range prompt gamma distribution generated from the planning CT.

One group used a slit camera detector system to validate their predictions of the proton range with dual-energy CT vs. single-energy CT, taken daily from patients undergoing proton treatment for prostate cancer [54]. They reported that the integration of prompt gamma imaging adds about 1 min per treatment field to the clinical workflow and that dual-energy CT-based predictions of the stopping power ratio agree with prompt gamma imaging more than single-energy CT-based predictions.

### 4.3. Ionoacoustics

The heat generated from the energy losses from the generated protons causes thermal expansion of the tissues. This leads to thermoacoustic emissions at a practically instantaneous timescale. The measurement of these waves by ultrasound, known as ionoacoustics, to map dose delivery is also an area of active investigation [55].

## 5. Image Guidance for Upright Treatment

A significant portion of the cost of treatment to construct a proton facility is due to the gantry that allows for different beam angles [56]. The large size of the gantry and the building required to house it makes up the majority of the cost.

One alternative is the use of a fixed horizontal beam line with a rotating chair positioning system, which may reduce the costs as it is less expensive to move the patient in the beam line vs. the beam around the patient. Previously constructed fixed beam lines are being repurposed by removing the old treatment chairs and installing new upright patient positioning systems [57], which allow for more flexibility in the treatment angles. Orthogonal kV X-rays can be received during pretreatment, similarly to receiving the treatment lying down [58].

Although CT simulations may be performed in the lying position and the images converted to a supine position for planning [59], there are significant deformations in the anatomy from a lying position than when treated upright, due to the relative change in direction of gravity. Thus, a dedicated CT scanner for upright daily image guidance is preferred for accurate treatment. Various models exist at certain proton centers and include scanners above the patient that descend for imaging alongside scanners that are mounted on columns and rotate around the patient, allowing for an isometric setup. Proton CT for daily setup is facilitated by the fixed beamline and rotating patient chair. For further reading, Volz et al. provided an excellent review of upright patient setup and guidance [60].

## 6. FLASH RT

The FLASH effect is defined as a decrease in radiation-induced normal tissue toxicities with dose delivery at ultra-high dose rates, compared to conventional dose rates used clinically [61]. Treatment of the first patient with FLASH-RT was delivered with electrons to a cutaneous T-cell lymphoma with favorable outcomes for tumor control and toxicity [62]. Groups are working on delivering FLASH-rate protons in clinics [63].

A recent trial has shown the feasibility of using FLASH protons in patients [64]. In this trial, the Bragg peak was placed distal to the patient, causing this beam to behave like a photon beam while in the patient, meaning that there was no sharp dose buildup or falloff.

The very short delivery times for FLASH would decrease the need for intrafraction motion management using conventional dose rates. Conversely, a precise pretreatment setup verification is required to ensure that the treatment is delivered to the intended target [65]. The slow acquisition time of CBCT would limit its utility for real-time imaging. Thus, MRI can provide information such as oxygenation and inflammation pre- and post-treatment, which may be useful for biological modeling [65]. One group has developed an inverse planning tool to optimize IMPT to pull the Bragg peak into the target volume and achieve FLASH dose rates in silico [66]. The higher dose-per-pulse rate of FLASH improves the ionoacoustic signal-to-noise ratio, which has led to an increased interest in this technology [65].

## 7. Conclusions

The importance of image guidance for proton therapy is clear due to the dosimetric properties of protons. Advances in image guidance in this space have generally followed advances in photon image guidance, possibly due to the much greater numbers of linear accelerators vs. proton gantries. However, as the number of proton gantries increases (119 gantries operational as of January 2023 with 37 under construction and another 29 in planning as of March 2023 [67]) and the number of patients treated with protons grows, it is important that there are continued advancements in image guidance for proton therapy so that it can fulfill its potential dosimetric benefits.

## Figures and Tables

**Table 2:** Methods of Pretreatment Image Guidance.

Type	Method	Advantages	Disadvantages
kV planar XR	kV X-rays are taken from the nozzle and compared to DRRs acquired at time of simulation or fiducial markers	Simple Able to visualize bony anatomy or radio-opaque visual markers	Inferior soft tissue contrast May not allow for direct visualization of the target Limited information on changes in tissue proximal to target
CT on Rails	Patients are imaged at a separate CT scanner before being moved on rails to the gantry couch, which has been registered to the CT scanner couch	Avoidance of imaging artifacts associated with CBCT Preferred in treatment rooms that cannot fit the CBCT system Reduces time that gantry is used for imaging	Additional ionizing radiation Possible additional movement if moving patient from CT scanner to gantry Additional time to move couch from CT to gantry No possibility of real-time guidance
CBCT	CBCT is placed on a gantry, nozzle, or robotic C-arm which rotates around the patient on the gantry couch	Additional soft tissue visualization compared to planar kV XR Allows for adaptive planning	Additional ionizing radiation Imaging artifacts No possibility of real-time guidance
MRI	Patient lies in a specially constructed MRI scanner which allows for proton beam delivery either in-line or perpendicular to magnetic field	No additional ionizing radiation Superior soft tissue contrast Possibility of real-time guidance	Not currently available clinically Complex deflections of proton beam and dose due to magnetic field Older pacemakers may not be MRI-compatible
Surface Tracking	Systems of visual cameras are installed in the gantry rooms and monitor moves in markers placed on the patient’s surface or the patient’s skin.	No additional ionizing radiation Real-time monitoring Pre- and intrafraction patient monitoring Can be complementary to CBCT Verification of nozzle setup	Surface movement does not always correspond to target movement Decreased accuracy in area of flat patient surface
